# Multi-level regulation of coelimycin synthesis in *Streptomyces coelicolor* A3(2)

**DOI:** 10.1007/s00253-019-09975-w

**Published:** 2019-06-27

**Authors:** Bartosz Bednarz, Magdalena Kotowska, Krzysztof J. Pawlik

**Affiliations:** Ludwik Hirszfeld Institute of Immunology and Experimental Therapy, Polish Academy of Sciences, ul. Rudolfa Weigla 12, 53-114, Wroclaw, Poland

**Keywords:** Actinomycetes, Coelimycin, *Streptomyces coelicolor*, Secondary metabolism, Antibiotics, Type I polyketide synthase

## Abstract

Despite being a yellow pigment visible to the human eye, coelimycin (CPK) remained to be an undiscovered secondary metabolite for over 50 years of *Streptomyces* research. Although the function of this polyketide is still unclear, we now know that its “cryptic” nature is attributed to a very complex and precise mechanism of *cpk* gene cluster regulation in the model actinomycete *S. coelicolor* A3(2). It responds to the stringent culture density and timing of the transition phase by the quorum-sensing butanolide system and to the specific nutrient availability/uptake signals mediated by the global (pleiotropic) regulators; many of which are two-component signal transduction systems. The final effectors of this regulation cascade are predicted to be two cluster-situated *Streptomyces* antibiotic regulatory proteins (SARPs) putatively activating the expression of type I polyketide synthase (PKS I) genes. After its synthesis, unstable, colorless antibiotic coelimycin A reacts with specific compounds in the medium losing its antibacterial properties and giving rise to yellow coelimycins P1 and P2. Here we review the current knowledge on coelimycin synthesis regulation in *Streptomyces coelicolor* A3(2). We focus on the regulatory feedback loop which interconnects the butanolide system with other *cpk* cluster-situated regulators. We also present the effects exerted on *cpk* genes expression by the global, pleiotropic regulators, and the regulatory connections between *cpk* and other biosynthetic gene clusters.

## Introduction

*Streptomyces* are Gram-positive, filamentous bacteria that are potent producers of secondary metabolites—specialized compounds with adaptive functions (Traxler and Kolter 2015)—many of which have antibiotic, immunosuppressant, antitumor, and other biological activities (Hopwood 2007). In the past two decades, the availability of complete genome sequences led to the development of over 20 biosynthetic gene cluster detection tools (www.secondarymetabolites.org/mining/) and revealed that the model organism *Streptomyces coelicolor* A3(2) could synthesize more than 20 secondary metabolites, many of them being still unidentified products of so-called cryptic or silent biosynthetic gene clusters (BGCs) (Bentley et al. 2002; Blin et al. 2017). Typical BGCs contain regulatory, tailoring, precursor supply, and transport genes organized around the main synthase subunit genes. In case of modular polyketide synthases and non-ribosomal peptide synthetases, they usually span over several tens of kilobases (Medema et al. 2015).

Among wide repertoire of *S. coelicolor* A3(2) chromosomally encoded bioactive molecules, there are 4 antimicrobial compounds: coelimycin A (CPK A, precursor of yellow coelimycins P1 and P2), calcium-dependent antibiotic (CDA), red-pigmented undecylprodigiosin (RED), and blue-colored actinorhodin (ACT) (Liu et al. 2013). Their production is induced by environmental, physiological, or nutrient limitation signals (Van Der Heul et al. 2018) coupled with vegetative mycelium autolysis and subsequent salvage of its constituents in order to form aerial mycelium that allows sporulation (Bibb 2005). Each biosynthetic gene cluster encodes its own pathway-specific *Streptomyces* antibiotic regulatory proteins (SARPs): CpkO (formerly KasO) and CpkN (*cpk* cluster), CdaR (*cda* cluster), RedZ and RedD (*red* cluster), and ActII-orf4 (*act* cluster) (Liu et al. 2013). Initially, regulatory roles of SARP cluster-situated regulators (CSRs) were believed not to extend beyond the borders of their respective metabolite biosynthetic gene clusters but this paradigm was shifted by mutational/overexpression studies suggesting that they may also control other BGCs indirectly by modulating global regulators such as AfsR2/AfsS (Huang et al. 2005). Nevertheless, it was found that cellular levels of *actII-orf4* and *redD* transcripts correlate with the production levels of respective secondary metabolites (Takano et al. 1992; Gramajo et al. 2014). Global (pleiotropic) regulators act on numerous, often distant genes in the chromosome and orchestrate multiple pathways to proceed with major cellular events such as morphogenesis, development, and antibiotic production. For many years, they have been believed to exert their functions on biosynthetic genes via cluster-situated regulators (McKenzie and Nodwell 2007) but later findings have demonstrated their ability to bind to promoters of biosynthetic genes (Ryding et al. 2002) or even within the coding sequences, implying their direct role in the regulation of secondary metabolism. In view of these findings, the definitions of “pathway-specific” and “pleiotropic” regulators as well as “higher-level” and “lower-level” may need revision. Until today, products of more than 50 genes were identified to directly or indirectly affect secondary metabolite production in *S. coelicolor* A3(2), most of them acting on multiple biosynthetic pathways (Van Wezel and McDowall 2011; Van Der Heul et al. 2018).

Biosynthetic gene coding for coelimycin type I polyketide synthase (PKS I) was first identified in 1997 by DNA probe hybridization to acyltransferase domain specific for malonyl-CoA (Kuczek et al. 1997). *S. coelicolor* A3(2) genome sequence publication in 2002 allowed to annotate *cpk* cluster (Pawlik et al. 2007). It wasn’t until 2010 when its products were detected as a yellow pigment excreted to the medium (yCPK) (Gottelt et al. 2010; Pawlik et al. 2010) later identified as coelimycins P1 and P2 (Gomez-Escribano et al. 2012), and mycelium-associated, colorless, antimicrobial compound (abCPK) (Gottelt et al. 2010) deduced to be coelimycin A (Challis 2014). As a matter of fact, biosynthesis of the yellow pigment was first reported in 1978 by Rudd in his PhD dissertation. In his work, Rudd successfully mapped the genetic locus responsible for the synthesis of the compound (Rudd 1978). CPK production is dependent on the medium composition and the density of the inoculum (Gottelt et al. 2010; Pawlik et al. 2010) which can be attributed to both the transcription regulation and participation of some molecules from the medium in the final biosynthetic steps. Expression of *cpk* genes at the very early transition phase of culture growth is an early event of the metabolic switch from primary to secondary metabolism (Nieselt et al. 2010).

Up to now, “big data” from numerous transcriptomic, proteomic, and chromatin immunoprecipitation (ChIP) experiments have provided fragmentary and scattered information about *cpk* cluster regulation in the overwhelming picture of *S. coelicolor* A3(2) secondary metabolism regulatory networks. The aim of this work is to extract, summarize, and comment on this information from the perspective of coelimycin synthesis regulation.

### Regulation within the *cpk* cluster

Originally the *cpk* cluster annotation was limited to *SCO6269–SCO6288* genes (Pawlik et al. 2007). Currently, the neighboring genes *SCO6265–SCO6268* coding the butanolide system proteins are also included in the cluster. The 58-kb coelimycin biosynthesis *cpk* cluster contains 24 genes functionally belonging to 5 groups: core biosynthetic (*cpkA*, *cpkB*, *cpkC*, *scoT*), precursor supply (*cpkPα*, *cpkPβ*, *accA1*, *cpkK*), post-polyketide tailoring (*scF*, *cpkD*, *cpkE*, *cpkG*, *cpkH*, *cpkI*), export (*cpkF*), and regulatory (*scbR*, *scbA*, *scbB*, *orfB*, *cpkO*, *scbR2*, *cpkN*) genes (Pawlik et al. 2007; Gomez-Escribano et al. 2012). Two genes (*cpkJ*, *cpkL*) have not been assigned a function. Eight transcriptional units (*cpkPβ/cpkPα/accA1*, *scF*, *cpkA/cpkB/cpkC*, *cpkD/cpkE/cpkF/cpkG/cpkO/cpkH*, *cpkI*, *cpkJ/cpkK/cpkL*, *scbR2*, *scoT/cpkN*) (Chen et al. 2016) and ten promoter regions binding different transcription factors have been identified (*pscbR*, *pscbA*, *porfB*, *paccA1/pscF*, *pcpkA/pcpkD*, *pcpkO*, *pcpkI/pcpkJ*, *pscbR2/pscoT*, *pcpkN*) (Takano et al. 2001; Takano et al. 2005; Gottelt et al. 2010; Li et al. 2015) but more promoters are predicted to be found in the intergenic regions of *cpk* cluster. Transcription start sites corresponding to transcripts of *scbR*, *scbA*, *cpkA*, *cpkC*, *cpkD*, *cpkI*, *cpkO*, *cpkH*, and *scbR2* have been determined (Takano et al. 2001; Romero et al. 2014; Jeong et al. 2016).

The core polyketide chain of coelimycin is assembled by the main subunits of the modular polyketide synthase—CpkA, CpkB, and CpkC (Gomez-Escribano et al. 2012). During synthesis, type II thioesterase ScoT maintains PKS activity by removal of non-reactive acyl residues blocking the “assembly line” and was shown to be mandatory for coelimycin synthesis (Kotowska et al. 2014). It was proposed that the intermediate is released from PKS as a hydroxyaldehyde and subsequently transformed by post-polyketide tailoring enzymes and presumably transported outside of the bacterial cell by CpkF membrane efflux protein where it undergoes epoxidation to coelimycin A. Its weak antibiotic activity can be attributed to two reactive epoxide rings. Spontaneous reactions of the epoxides with N-acetylcysteine or glutamate present in the medium lead to formation of yellow-pigmented coelimycins P1 or P2, respectively (Gottelt et al. 2010; Gomez-Escribano et al. 2012). It is likely, that other coelimycins, also colorless, are formed as a result of the reaction of the epoxide rings with other substrates.

The functions of the regulatory proteins encoded within *cpk* cluster are as follows. ScbA is a γ-butyrolactone (GBL) synthase (Hsiao et al. 2007) accompanied by ScbB which also participates in GBL synthesis (Sidda et al. 2016). ScbR is a TetR-like GBL receptor, its homolog ScbR2 is a pseudo-GBL receptor not affected by GBLs, but shown to bind RED, ACT, and other antibiotics (Xu et al. 2010a; Wang et al. 2014). OrfB is a homolog of histidine protein kinases (Takano et al. 2005) but its target for phosphorylation has not been found. CpkO and CpkN are two SARP proteins. Their binding sites are not known. CpkO is an activator necessary for *cpk* gene expression as shown by transcriptomic and qRT-PCR analysis of *ΔcpkO* mutant strain (Gottelt et al. 2010; Takano et al. 2005). Since SARPs generally activate their target genes, CpkN is also likely an activator.

If the conditions of growth of wild-type *S. coelicolor* A3(2) are suitable for CPK production, the yellow pigment is observed about 24 h earlier than the typical time when other colored metabolites appear (Gottelt et al. 2010; Pawlik et al. 2010). In a high-resolution time-series transcriptomic study of a fermenter-grown culture in minimal medium with glucose and glutamate, all the *cpk* cluster genes showed a strong transient expression peak around 22–24 h and many of them remained at constantly elevated expression levels afterwards (Nieselt et al. 2010). Transcription peaks of regulatory genes *scbR*, *scbA*, *scbB*, and *cpkO* (22 h) preceded those of other *cpk* cluster genes. Next was *orfB* (peak at 23 h time point) followed by the rest of the *cpk* cluster genes including regulators *scbR2* and *cpkN* (24 h). Transcription of *scoT* needed to maintain the enzymatic activity of the modular PKS subunits started to increase 1 h later than that of core biosynthetic genes. Interestingly, this sharp peak of *cpk* cluster regulatory gene transcription preceded the moment traditionally seen as the onset of secondary metabolism correlated with phosphate depletion and upregulation of the *pho* regulon (35 h in the same study) followed by the expression of *red* (38 h) and *act* (43 h) gene clusters (Nieselt et al. 2010).

**Fig. 1:**
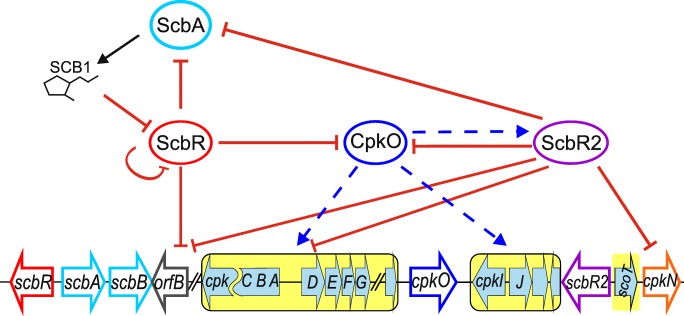
Coelimycin biosynthetic gene cluster regulation by the cluster-situated regulators. CPK biosynthetic genes are marked with a yellow background. The lines ending with arrows indicate transcription activation, with the exception of an arrow indicating γ-butyrolactone SCB1 production. The lines ending with bars indicate repression of transcription or inhibition of ScbR by SCB1. The solid lines indicate promoter binding while dashed lines imply an indirect or unknown regulatory mechanism. See the text for further information and references

The rapid increase and sharp decline of the transcription of *cpk* genes are governed by the butanolide system (Fig. 1). During the exponential-growth phase, the synthesis of γ-butyrolactone SCB1 (by ScbA protein) and its receptor protein ScbR is on the basal level. At this time point, ScbR exists mainly as a DNA-protein complex—it binds promoter regions of *scbR*, *scbA* (Takano et al. 2001), *cpkO* (Takano et al. 2005), and *orfB* (Li et al. 2015) genes, acting as a transcription inhibitor. The SCB1 level rises proportionally to the number of dividing bacterial cells. After its concentration reaches the threshold, SCB1 binding to ScbR results in its dissociation from DNA and subsequent derepression of its own, *scbA* and that of *cpkO* gene expression (Takano et al. 2001; Takano et al. 2005). The elevated level of CpkO directly or indirectly activates the transcription of *cpk* genes, including that of *scbR2* encoding pseudo-GBL receptor*.* Inhibitory interaction of ScbR2 with *scbA* promoter consequently blocks SCB1 biosynthesis. Since ScbR2 is known to bind to *cpkO*, *cpkN*, and *orfB* promoters and to inhibit their transcription (Gottelt et al. 2010; Li et al. 2015), it has been proposed that it may serve as a switch turning off coelimycin synthesis.

A number of deletion mutants of the butanolide system genes were analyzed, leading to the following observations: (i) in Δ*scbA* mutant transcription of *scbR*, *scbR2*, *cpkO*, and *scbA* is diminished (D’Alia et al. 2011) and addition of exogenous SCB1 to Δ*scbA* mutant restores transcription of both *scbR* and *scbR2* but not *scbA* (Takano et al. 2005), (ii) in *ΔcpkO* mutant transcription of *scbR2* is diminished (Gottelt et al. 2010), (iii) *∆scbR2* mutant is upregulated in *scbR*, *scbA*, (Wang et al. 2011; Li et al. 2015) and *cpkO* transcription (Gottelt et al. 2010), (iv) in Δ*scbR* mutant expression of *scbR2* (Gottelt et al. 2010) and *cpkO* (Takano et al. 2005) is constitutive, transcription of *scbR* is upregulated and transcription of *scbA* abolished (Takano et al. 2001; Li et al. 2015).

Inactivation of either *scbA* or *scbR* was shown to abolish SCB1 production (Takano et al. 2001). Addition of exogenous SCB1 to *ΔscbA* mutant-restored transcription of *scbR*, but not that of *scbA* which led to a proposal of these proteins forming a complex required to activate *scbA* transcription (Takano et al. 2005; Mehra et al. 2008). In an excellent review paper, it was speculated that ScbR, although being an autorepressor and the repressor of *cpkO*, may act as an activator upon binding to *scbA* promoter and that the concentration of exogenous SCB1 is high enough to saturate all ScbR molecules thus preventing activation of *scbA* transcription (Van Wezel and McDowall 2011). Our explanation for the mandatory (“activatory”) role of ScbR in *scbA* transcription follows (see Fig. 1).

In *scbR* deletion mutant, there is no ScbR necessary for *cpkO* repression. Upregulated CpkO may raise the concentration of ScbR2 protein which is a repressor of *scbA* (Wang et al. 2011). The same mechanism may account for upregulation of *scbR/scbR2* and failure to restore *scbA* transcription in *scbA* null mutant by exogenous SCB1 (Takano et al. 2005). Concentration of exogenous SCB1 is probably high enough to complex whole cellular ScbR bound to *scbR* and *cpkO* promoters (thus creating conditions similar to those in ∆*scbR* mutant) resulting in derepression of both genes. This leads to upregulation of *scbR2* transcript level by CpkO and repression of *scbA* by ScbR2. The reason for abolished transcription of *scbA* in *ΔscbA* mutant may be simply repression of its promoter by ScbR devoid of SCB1.

Not assigning ScbR an activatory role for *scbA* transcription but rather attributing its reduction to the upregulated ScbR2 level as an indirect consequence of *scbR* deletion/inactivation allows to provide a mechanism accounting for seemingly contradictory observations (Fig. 1). It would be intriguing to unravel the exact pathway (direct or non-direct) of CpkO activatory impact on *scbR2* transcription.

### Influence of the *cpk* cluster-situated regulators on other metabolic pathways

The butanolide ScbA/ScbR/ScbR2 system was thought to primarily target coelimycin biosynthetic gene cluster in *S. coelicolor* A3(2) but the turning point was publication of 16 and 58 genome-scale, confirmed binding sites of ScbR and ScbR2, respectively, along with their respective null mutants’ transcriptomic data. It revealed that 30.1% and 42.3% of all genes are at least 20% differentially expressed in *∆scbR* and *∆scbR2*, respectively, in comparison with the parent strain M145 (Li et al. 2015). Taken together, these data shifted ScbR and ScbR2 impact range from pathway-specific to pleiotropic regulators. It also provided experimental proof for the key role of ScbR2 in molecular cross-regulation of secondary metabolite synthesis.

Mutants ∆*scbA* and ∆*cpkO*, which both lack ScbR2 synthesis, showed precocious *actII*-*orf4* and *redD* transcription but only the former strain was accordingly advanced in production of ACT and RED (Takano et al. 2001; Gottelt et al. 2010; D’Alia et al. 2011). No obvious ACT and RED synthesis phenotype was observed in the ∆*cpkO* strain (Takano et al. 2005). This discrepancy can be attributed to different levels of ScbA protein in both mutants. The lack of ScbR2 may be the cause of precocious *scbA* transcription in ∆*cpkO* (Takano et al. 2005) but it cannot affect the complete lack of ScbA protein in the mutant ∆*scbA* for the absence of the gene. Deletion of *scbA* was indeed associated with earlier expression of the genes encoding primary metabolism enzymes that ensure antibiotic precursor supply (D’Alia et al. 2011) required for ACT and RED synthesis.

CPK overproducing ∆*scbR2* strain was dramatically reduced in ACT, RED, and CDA synthesis. This phenotypic effect was reflected in transcript levels of respective activators (increased *cpkO*, decreased *actII-orf4*, *redZ*, *cdaR*), all of which were shown to be direct targets for binding by ScbR2 (Li et al. 2015). As discussed earlier, ScbR2 is a repressor of *cpkO* gene; hence, the effect of *scbR2* deletion on transcription of ACT, RED, and CDA activators is surprising, suggesting a potential activatory role of ScbR2 or another regulatory mechanism. Such a mechanism would downregulate the transcription of *actII-orf4*, *redZ*, and *cdaR* perhaps in response to ∆*scbR2-*uninhibited coelimycin production or highly elevated levels of *scbA* transcription in the mutant (Wang et al. 2011). ScbR2 DNA-binding activity was found to be relieved upon binding of RED and ACT further underscoring its antibiotic cross-regulatory role (Xu et al. 2010a). As for now, CPK binding to ScbR2 wasn’t shown in *S. coelicolor* A3(2) but studies conducted in *S. lividans* TK24 suggest the existence of such interaction (Sun et al. 2017).

An important target repressed by both ScbR and ScbR2 is *accA2* gene (Li et al. 2015). AccA2 is an essential subunit α of acyl-CoA carboxylases, enzymatic complexes providing carboxylated precursors for fatty acid and polyketide biosynthesis. Its close homolog, AccA1 encoded within the *cpk* cluster, was shown by in vitro reconstitution experiment to cooperate with the same acyl-CoA carboxylase β subunits as AccA2 (Rodríguez and Gramajo 1999; Rodríguez et al. 2001). CpkK is another putative β subunit of acyl-CoA carboxylases, homologous to an essential AccB protein (Rodríguez et al. 2001). It is possible that CpkK may form complexes with both AccA1 and AccA2.

ScbR and ScbR2 proteins were also shown to interact forming a heterodimer able to bind a novel target, the promoter of *SCO5158*, a gene involved in metal transport. The heterodimer was also shown to coexist with ScbR and ScbR2 homodimers which is how these proteins most commonly bind DNA (Li et al. 2017).

### Effects of pleiotropic regulators on coelimycin synthesis

**Fig. 2:**
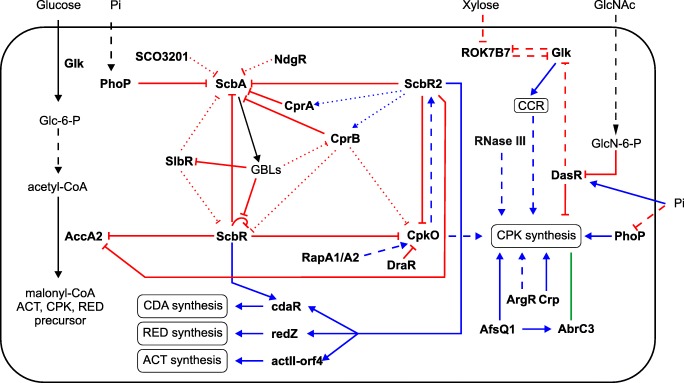
The regulatory pathways interconnected with *cpk* cluster regulation. The diagram links nutrient signals with pleiotropic and pathway-specific regulators affecting coelimycin synthesis and the feedback effect of *cpk* cluster genes. The blue lines ending with an arrow indicate activation. The red lines ending with a bar indicate repression. The green line with no ending indicates evidence of DNA binding, but the effect on transcription is unknown. The solid lines indicate the direct effect (DNA or ligand binding), dashed lines indicate indirect effect, and dotted lines indicate proposed but direct interactions. The thin black lines indicate transport or transformation of chemical compounds. Only the effects of butanolide system proteins ScbR and ScbR2 on other biosynthetic gene clusters were included in the diagram as they most accurately represent the impact of *cpk* cluster transcription on the production of other antibiotics. CCR–carbon catabolite repression. See the text for further information and references

The network of interactions between the *cpk* gene cluster, butanolide system, and other regulatory proteins of *S. coelicolor* A3(2) is outlined in Fig. 2.

### Two-component systems

Two-component systems (TCSs) are key signal transduction mechanisms in bacteria, acting as sensors of environmental condition changes and response modulators of transcription. These functions are accomplished through their membrane-bound histidine kinases (HKs) and response regulators (RRs), respectively (Rodríguez et al. 2013). *S. coelicolor* A3(2) genome encodes an impressive number of 67 typical TCSs, of which only a few have been shown to regulate secondary metabolite synthesis.

#### AfsQ1/Q2

AfsQ1/Q2 two-component system is involved in the regulation of carbon, phosphate, and nitrogen metabolism along with antibiotic synthesis. AfsQ1 was shown to activate synthesis of ACT, RED, and CDA by directly binding to the promoters of *actII-orf4*, *redZ* (but not *redD*), and *cdaR* (Wang et al. 2013). Deletion of *afsQ1/Q2* revealed a dramatically decreased expression of *cpk* cluster and abolished CPK synthesis. AfsQ1 binds to the *cpkA* promoter and not to any other promoter in the cluster, including that of *cpkO* or *cpkN*. Interestingly, when AfsQ1 DNA-binding motif was mutated, CPK synthesis was abolished as a result of the decrease in *cpkA/B/C* transcript; however, expression of other *cpk* genes was enhanced. Authors concluded that AfsQ1 binding to the *cpkA* promoter may help recruit RNA polymerase and activate *cpkA/B/C* transcription. They also suggested that another regulatory cascade mechanism is involved in regulation of *cpk* cluster by AfsQ1/Q2 (Chen et al. 2016).

#### DraR/K

DraR/K TCS regulates physiological and morphological differentiation in *S. coelicolor* A3(2) along with secondary metabolism in a medium-dependent manner (Yu et al. 2012; Yu et al. 2014). DraR represses coelimycin and undecylprodigiosin and activates actinorhodin production. Its regulatory role in CPK and ACT biosynthesis was shown to be mediated by binding to *cpkO* and *actII*-*orf4* promoters, respectively. On the contrary, DraR regulates RED synthesis independently of *redD* or *redZ* promoter binding. A possible cooperation of DraR with AfsQ1 was proposed in the regulation of *actII*-*orf4*, adding another level of complexity to secondary metabolism coordination in *S. coelicolor* A3(2) (Yu et al. 2012). Transcriptomic studies revealed a general drastic upregulation of *cpk* cluster genes in ∆*draR/K* mutant over 60 h time. The effect was most pronounced in 42 h time point and involved regulatory genes *cpkO* and *scbR2*. Butanolide system genes *scbR*, *scbA*, and *scbB* transcripts were upregulated in 36 h and 42 h time points but showed downregulation after 60 h in the mutant when compared with the parent strain. The decrease in *scbA* and *scbR* transcript abundance in 60 h time point might be a consequence of *scbR2* transcription, which peaks after 42 h and decreases until 60 h time point (Yu et al. 2014). Concluding from the available data, DraR/K two-component system is a *cpk* cluster repressor.

#### PhoR/P

PhoR/P two-component system senses phosphate starvation and suppresses central metabolism and development and secondary metabolism until enough phosphate is salvaged for morphological differentiation. From ChIP on chip and transcriptomic studies, PhoP was shown to bind hundreds of genomic regions and affect transcription of corresponding genes. The majority of target genes are repressed rather than activated by PhoP. Downregulation of all detected *cpk* cluster transcripts in *∆phoP* mutant clearly suggested that PhoP is a *cpk* cluster activator. Interestingly, *cpk* cluster was found to be the most PhoP-enriched region of *S. coelicolor* A3(2) genome due to intensive binding of the regulator within coding sequences of *cpkB* (2 sites) and *cpkC* (1 site) but not in promoter regions. PhoP was also shown to bind to *scbA* promoter and the observed enhancement of *scbA* transcription in ∆*phoP* mutant led the authors to the conclusion that it is *scbA* repressor (Allenby et al. 2012). In another study, *scbR* transcript and protein were more abundant in ∆*phoP* than in the wild-type strain (Thomas et al. 2012). To our knowledge, decreased transcription of *cpk* genes and at the same time upregulation of *scbA* and *scbR* transcripts in ∆*phoP* mutant could be a consequence of drastically downregulated *scbR2* transcription that was indeed observed (Allenby et al. 2012).

#### RapA1/A2

Deletion of *rapA1/A2* did not cause any change in growth or morphology of *S. coelicolor* A3(2); however, it reduced ACT production. Further proteomic studies revealed decreased abundance of CpkI protein in the *∆rapA1/A2* mutant. Reverse-transcriptase PCR showed downregulation in both pathway-specific regulatory genes *actII-orf4* and *cpkO* transcripts providing a reason for reduced ACT production and indicating downregulation of coelimycin synthesis in *∆rapA1/A2* mutant, although the product of *cpk* cluster was not characterized at that time (Lu et al. 2007).

#### AbrC1/C2/C3

AbrC1/C2/C3 is an atypical TCS comprised of two histidine kinases AbrC1/C2 and a response regulator AbrC3. The system is highly conserved among all sequenced *Streptomyces* species. AbrC3 is an activator of morphological development along with ACT, RED, and CDA production (Yepes et al. 2011). It was shown to bind to *actII-orf4* but not to *redD* or *redZ* promoters. AbrC3 intragenic binding to *cpkC* was detected, although no significant changes in the expression of *cpk* genes was demonstrated (Rico et al. 2014). Interestingly, AbrC3 is positively regulated by AfsQ1 through the direct binding in the promoter region (Wang et al. 2013). Further studies using different growth conditions may be necessary to induce the regulatory effect of AbrC1/C2/C3 on coelimycin synthesis.

### TetR family and γ-butyrolactone binding proteins

TetR family regulators are widely distributed among bacteria. They are transcription repressors with a helix-turn-helix DNA-binding motif that dissociate from their target sequences and derepress them upon ligand binding. The ligand molecules are often quorum-sensing signaling compounds and end-products or intermediates of antibiotic biosynthetic pathways. *S. coelicolor* A3(2) genome encodes at least 150 TetR-like regulators (Ramos et al. 2005); two of which, ScbR and ScbR2 were described earlier in this work.

#### SCO3201

*S. coelicolor* A3(2) TetR family SCO3201 protein was identified in an attempt to find a major repressor of ACT synthesis in *S. lividans* that accounts for its very weak actinorhodin production capacity. *SCO3201* gene disruption in *S. coelicolor* A3(2) did not produce any distinct phenotype. However, it was demonstrated to inhibit ACT, RED, and CDA synthesis along with morphological differentiation and sporulation upon protein overexpression. SCO3201 is a negative regulator and was shown to bind to the promoter region of *scbA* but not that of *scbR* or *cpkO*. SCO3201 overexpression led to downregulation of *scbR* and *cpkO* transcription in exponential but not in the stationary growth phase while the transcription of *scbA* was upregulated in the stationary growth phase. Authors suggested that SCO3201 may lose its repressory activity upon binding an unknown ligand in the stationary phase (Xu et al. 2010b). We hypothesize that SCO3201 binding to *scbA* promoter is repressive (Fig. 2) and leads to decreased SCB1 production in the exponential phase and in turn—decreased *scbR*, *cpkO*, and *scbR2* transcripts. After dissociation of SCO3201 from *scbA* promoter in the stationary phase, low abundance of ScbR2 production is insufficient to fully repress *scbA*. In order to gain a better view of the regulation mechanism, it would be interesting to measure the *scbR2* expression pattern in the same conditions. Functionally, SCO3201 might mimic ScbR; thus, it would be intriguing to assess its GBL binding activity.

#### CprA/CprB

CprA and CprB share 90.7% amino acid sequence identity and they are close homologs of ScbR. CprA was shown to be the activator of sporulation and ACT and RED production, while CprB was demonstrated to inhibit sporulation and ACT synthesis but had no effect on RED production (Onaka et al. 1998). Interestingly, CprA and CprB were found to repress *scbA* promoter and, consequently, GBL production (Li et al. 2015). Binding of CprB to the promoters of *scbR* and *cpkO* was also demonstrated. In the same studies, addition of the extract from *S. coelicolor* A3(2) liquid culture but not the exogenous SCB2 γ-butyrolactone caused dissociation of CprB from an artificially synthesized consensus sequence (Bhukya et al. 2014). Interestingly, the promoter of *cprA* was bound by ScbR and ScbR2 while the promoter region of *cprB* was bound by ScbR2, suggesting the existence of a feedback loop (Li et al. 2015).

#### SlbR

SlbR does not belong to any characterized regulator family but is capable of binding γ-butyrolactone SCB1. It was shown to bind to the promoter of *scbR* and *scbA*, and the DNA binding was relieved in the presence of SCB1, but no profile of butanolide system and *cpk* cluster gene transcription was provided. Pattern of expression of *slbR* gene resembles that of *scbR* but contrary to ScbR; SlbR binds *adpA* promoter and affects morphology (Yang et al. 2012). It represses ACT and RED synthesis along with spore formation in rich media. Its properties make it more similar to ArpA, ScbR homolog in *S. griseus.* Further studies are anticipated to reveal the pleiotropic effects of SlbR on secondary metabolism and differentiation.

### ROK-signature proteins

Glk and Rok7B7 belong to the ROK (regulators, ORFs, and kinases) family of proteins that contains transcriptional factors and kinases associated mainly with primary metabolism. However, both Glk and Rok7B7 were shown to affect not only carbon catabolite repression (CCR) but also growth and secondary metabolism (Świątek et al. 2013; Romero-Rodríguez et al. 2016).

#### Glk

Glucose kinase (Glk, GlkA) plays a major role in carbon catabolite repression (CCR), a mechanism ensuring sequential utilization of carbon sources, from the most to the least preferred, when a mixture of them is available in the cell environment (Titgemeyer and Brückner 2002). Glk has two distinct activities: (i) enzymatic (phosphorylation of glucose to glucose-6-phosphate which can enter central carbon catabolism pathways) and (ii) regulatory (possible interactions with transcription factors) (Gubbens et al. 2012). In order to distinguish between those 2 effects of *S. coelicolor* A3(2) Glk, *glkA* null mutant complemented with heterologous Glk from *Zymomonas mobilis*, which complements only enzymatic activity, was constructed (ScoZm strain) (Romero-Rodríguez et al. 2016). Transcriptomic profiles of *S. coelicolor* A3(2) parent strain on media with both glucose and agar (repressive conditions) or agar as the sole carbon source (non-repressive conditions) were compared. Moreover, transcriptomic profiles of ScoZm and the parent strain grown in repressive conditions were analyzed. The putative kinase *orfB* and *cpkN* transcripts were downregulated, and that of *scbA* was upregulated in ScoZm when compared with the M145 wild-type strain. These effects were not observed in M145 grown in repressive relative to non-repressive conditions, suggesting the dependence of these genes on the regulatory activity of GlkA. On the other hand, the presence of glucose upregulated transcription of genes *scF–cpkG (SCO6272–SCO6279)* and *cpkI–cpkL (SCO6282–SCO6285)* in the wild-type strain. Interestingly, the effect of glucose on *cpk* cluster expression depends on other constituents of the medium—in minimal medium NMMP (Romero-Rodríguez et al. 2016)—addition of glucose enhanced CPK synthesis, while on rich medium 79, it was prevented (Pawlik et al. 2010). It was also reported that different additional carbon sources in the NMMP medium—mannitol or fructose—can elicit contrary effects on *cpk* cluster protein levels upon glucose addition—activation or repression, respectively (Gubbens et al. 2012).

#### Rok7B7

Rok7B7 is a repressor of the xylose operon and an activator of the sugar phosphotransferase system (PTS) genes, involved among others in N-acetylglucosamine (GlcNAc) internalization. The protein was shown to be a direct repressor of RED production and an ACT synthesis activator (Park et al. 2009; Świątek et al. 2013). The only trace information on Rok7B7 regulatory effect on coelimycin synthesis is found in proteomic studies, which demonstrated that CpkH protein is much less abundant in *∆rok7B7* mutant than in the parent strain (Świątek et al. 2013).

### Other regulators

#### DasR

DasR is a global regulator linking nutrient stress to antibiotic synthesis. High concentration of GlcNAc in minimal growth medium, resembling its accumulation after autolytic vegetative mycelium degradation in nature, triggers the onset of secondary metabolism. DasR also connects carbon, nitrogen, and phosphate metabolism. The protein dissociates from its targets upon binding of glucosamine-6-phosphate, an intermediate of GlcNAc metabolism (Rigali et al. 2006), while at the same time, high phosphate concentrations enhance its DNA-binding activity (Tenconi et al. 2015). DasR controls RED and ACT biosynthesis through the respective pathway-specific regulators. Indeed, DasR was shown to bind to the promoters of *redD*, *redZ*, and *actII-orf4* and repress them (Rigali et al. 2008; Świątek-Połatyńska et al. 2015). ∆*dasR* mutant showed more abundant *scbA* and *cpkO* transcripts which resulted in enhanced transcription of the *cpk* cluster, clearly showing that DasR is coelimycin synthesis repressor. The regulator was demonstrated to bind to intragenic regions of three biosynthetic genes *cpkA/B/C* but no direct interaction with *cpk* or butanolide system gene promoters was observed implying a more complex mechanism of *cpk* cluster regulation (Świątek-Połatyńska et al. 2015).

#### ArgR

In bacteria, ArgR is a transcriptional repressor of arginine and pyrimidine biosynthetic genes that uses l-arginine as co-repressor. However, it was also demonstrated to activate ACT and RED production (Pérez-Redondo et al. 2012). Interestingly, *∆argR* mutant exhibited the formation of spore-like chains when grown in liquid culture (Botas et al. 2018). ArgR was found to be an activator of butanolide system and *cpk* gene cluster. Together with post-PKS and precursor supply, regulatory genes *scbA*, *scbB*, *scbR*, *scbR2*, *cpkO*, and *cpkN* were all downregulated in *∆argR* mutant (Pérez-Redondo et al. 2012; Botas et al. 2018). *∆argR* mutant *cpk* cluster genes followed a profile of downregulation and delayed expression in the stationary phase, probably as a result of additional control mechanisms to those of ArgR (Botas et al. 2018).

#### Crp

Crp (cyclic AMP receptor protein) is a transcription regulator controlling colony development, precursor synthesis/flux, and production of at least 8 secondary metabolites in *S. coelicolor* A3(2) (Derouaux et al. 2005). Unlike in Gram-negative bacteria, Crp does not play a role in CCR in *Streptomyces* (Romero-Rodríguez et al. 2016). The regulator was demonstrated to activate ACT and CDA synthesis. *∆crp* mutant was hypersporulating and was noticeably delayed in germination, growth, and RED synthesis although ultimately produced the same amount of antibiotic as the parent strain (Derouaux et al. 2005; Gao et al. 2012). Indeed, Crp was shown to directly bind in or upstream of the coding regions of *actII-orf4*, *redZ*, and *cdaR* along with *cpkA* and some precursor supply and post-PKS *cpk* genes. The transcription of *accA1*, *scF*, and *cpkA* was upregulated upon Crp overexpression, suggesting its activatory role in coelimycin production in *S. coelicolor* A3(2) (Gao et al. 2012).

#### NdgR

NdgR (regulator for nitrogen source-dependent growth and antibiotic production) is a direct transcriptional activator of leucine and methionine biosynthesis (Kim et al. 2012); a glycerol utilization operon repressor (Lee et al. 2017); and a regulator of growth, morphological differentiation, and nitrogen source-dependent ACT production (Yang et al. 2009). It targets *scbR/A* promoter region. In proteomic studies CpkE and CpkJ were found to be more abundant in ∆*ndgR* mutant than in the parent strain (Yang et al. 2009); thus, we speculate that NdgR is *cpk* cluster inhibitor.

#### GntR

GntR (SCO1678) is a FadR subfamily gluconate-binding repressor of the gluconate operon *SCO1679*–*SCO1682*. ACT synthesis was shown to be decreased in gluconate-supplemented in comparison with the glucose-supplemented SMM medium in *S. coelicolor* A3(2). What is more, in *∆gntR* mutant, gluconate induced the production of coelimycin, suggesting a connection between gluconate metabolism and the production of this compound. The authors speculated the existence of an unknown protein acting as a switch from ACT and RED synthesis to minor or cryptic secondary metabolite production in the absence of alternative carbon sources and GntR protein (Tsypik et al. 2017).

#### RNase III

RNase III is a double-stranded RNA-specific endoribonuclease involved in rRNA processing, sense/antisense RNA degradation, and other gene expression regulation mechanisms (Blomberg et al. 1990; Babitzke et al. 1993; Drider and Condon 2004). *S. coelicolor* A3(2) *rnc* deletion mutant was unable to produce ACT, RED, and CDA in a set of different solid media and SMM liquid medium, optimized for antibiotic production (Sello and Buttner 2008). RNA-seq analysis of *S. coelicolor* A3(2) *rnc* mutant transcriptome revealed strong downregulation of *cpkD*, *cpkE*, *cpkF*, *cpkG*, *cpkO*, *cpkI*, *cpkJ*, and *cpkK* along with *cdaR* and other *cda* gene transcription in comparison with the wild-type strain. In the same study, RNase III was demonstrated to immunoprecipitate with mRNA of *cpkE* and *cpkI* transcripts suggesting RNase III can regulate these gene transcripts’ levels through mRNA binding without cleavage mechanism (Gatewood et al. 2012). A scenario has been proposed in which RNase III could bind and/or cleave the double-stranded mRNA-cutoRNA (convergent untranslated overlapping RNA—a species of antisense RNAs) pair that resulted from the overlap of 3′ untranslated regions from convergently oriented genes. The implication of this could be altered stability of one or both transcripts; however, the overall functions of RNase III and cutoRNAs are not clear yet (Moody et al. 2013). Beside one cutoRNA localized in *SCO6268*, other antisense RNAs have been identified in *cpk* cluster genes (*scbR*, *scbA*, *accA1*, *cpkC*, *cpkB*, *cpkE*, *cpkH*) along with potential small non-coding RNAs localized in the *cpk* intergenic regions (*scr6287–6286, scr6280*) (Moody et al. 2013; Romero et al. 2014), suggesting an additional regulatory mechanism to that of cluster-situated and global regulators.

### Coelimycin biosynthetic gene cluster as part of the global regulatory network

The physiological role of coelimycin in *S. coelicolor* A3(2) is not known. It is unlikely to be a “chemical weapon” against other microorganisms, as the weak antibiotic compound coelimycin A is unstable. We speculate that due to the presence of two reactive epoxide rings, coelimycin A may act as a detector of certain compounds in the environment and take part in an unknown signaling pathway. Whatever its biological function, it apparently requires stringent control and is released only transiently at the beginning of the metabolic switch from exponential growth to antibiotic production.

In view of the available data, we describe a consistent mechanism of coelimycin biosynthetic gene cluster regulation by the butanolide system proteins and CPK biosynthesis pathway-specific activator CpkO (Fig. 1). Binding sites of both SARPs from this cluster, CpkO and CpkN, have not been found so far. Finding differences between their direct targets might clarify why transcription of some *cpk* genes remains elevated after the sharp activation peak (Nieselt et al. 2010). Nevertheless it is clear that CpkO activates the synthesis of ScbR2 protein which in turn acts as a switch turning off the expression of *cpk* gene cluster. This connection (activation of *scbR2* by CpkO) allows to explain some previously unclear experimental results. Moreover, ScbR2 appears to be a key player in cross-regulation of other biosynthetic clusters. As opposed to being the transcription repressor of *cpk* cluster, it seems to activate ACT, RED, and CDA clusters (Li et al. 2015).

The coupling of *cpk* and butanolide system genes in the cluster and the existence of CpkO-ScbR2-ScbA feedback loop interconnecting *cpk* and butanolide system may indicate their co-evolution (Medema et al. 2014) and their co-influence on other biosynthetic gene clusters. The outcome of the interplay within the *cpk* cluster is especially important when we take into account that coelimycin production is an early secondary metabolism event in the life cycle of the cell and therefore, it is involved in shaping the rest of it. Indeed, both ScbR and ScbR2 were shown to participate in multiple regulatory pathways (Li et al. 2015). The impact of *cpk* cluster is also reinforced by the existence of only one GBL synthase (ScbA/ScbB), producing at least 8 different GBLs (Sidda et al. 2016), and several GBL-receptor homolog genes encoded in *S. coelicolor* A3(2) genome (Nishida et al. 2007). SlbR, a protein non-resembling typical GBL receptors, has also been demonstrated to bind GBL, further expanding the range of controlled pathways.

We made an attempt to bring together a diagram (Fig. 2) of pleiotropic regulatory systems that influence *cpk* cluster expression including the links to other antibiotic BGCs, but the different cross-regulatory connections were difficult to integrate. Some regulators are well characterized and their description is firmly based on different experimental techniques. Most of available information comes from transcriptomic and proteomic studies as well as genome-scale detection of binding sites. Differences in experimental conditions, data acquisition, and data analysis between omics-based studies make it difficult to compare their results and hinder the creation of a unified regulatory-network model.

Concerted action of global regulators is responsible for the ability of streptomycetes to utilize diverse and often fluctuating food sources. Nutrient limitation leads to vegetative mycelium autolysis and metabolic switch towards secondary metabolite production and morphological differentiation. Through the action of DasR, *S. coelicolor* A3(2) may sense the availability of GlcNAc (a preferred carbon and nitrogen source) and hold or induce secondary metabolism according to other signals indicating if the environment is rich in nutrients or not (Rigali et al. 2008). DasR, as well as other regulators involved in the intertwining nutrient-sensory networks, such as Glk, PhoR/P, AfsQ1/Q2, Crp (Urem et al. 2016) and many others, influences transcription of coelimycin biosynthetic gene cluster showing a connection between the nutrient sensing pathways and the butanolide system, although their exact interactions remain to be established. Data presented in this work suggest that pleiotropic regulators control *cpk* cluster on multiple levels by binding to promoters and affecting transcription of (i) the butanolide system genes, (ii) the *cpk* pathway-specific activator *cpkO* and (iii) the core biosynthetic genes. The reported observations of binding of global regulators within the coding sequences of core biosynthetic genes and emerging evidence of non-coding RNAs’ involvement in the control of secondary metabolism set new challenges in deciphering the multi-level regulation of secondary metabolite synthesis in *Streptomyces coelicolor* A3(2).
